# The effects of different tensile parameters for the neurodynamic mobilization technique on tricipital muscle wet weight and MuRf-1 expression in rabbits with sciatic nerve injury

**DOI:** 10.1186/s12984-015-0034-4

**Published:** 2015-04-15

**Authors:** Yan Wang, Ming Ma, Qiang Tang, Luwen Zhu, Melanie Koleini, Dequan Zou

**Affiliations:** Rehabilitation medicine center of the second affiliated hospital of Heilongjiang university of Chinese medicine, Harbin, 150001 China; Rehabilitation medicine college of Heilongjiang university of Chinese medicine, Harbin, 150040 China; Program in Physical Therapy, Washington University School of Medicine, St. Louis, MO 63110 USA

**Keywords:** Neurodynamic mobilization technique (NMT), Tricipital muscle wet weight, MuRf-1, Peripheral nerve injury

## Abstract

**Background:**

After peripheral nerve injury, muscles without innervation begin to undergo atrophy. Research has suggested that MuRf-1 may play a role in muscle atrophy. The neurodynamic mobilization technique (NMT) is a manual therapy method used to elongate a nerve along its long axis, resulting in improved blood flow to the nerve. However, the nerve can be damaged if elongated too much. The purpose of this study is to observe the effect of NMT on muscle wet weight and MuRf-1 expression in rabbits with sciatic nerve injury.

**Methods:**

Six adult rabbits were measured to determine the relationship between the joint angle of the lower limb and percent of sciatic nerve elongation to define the tensile parameters of NMT; Thirty adult rabbits were randomly assigned into a sham, model, NMT-A, NMT-B, or NMT-C groups. Four weeks post-treatment, the wet mass of the tricipital muscles and MuRf-1 expression were observed.

**Results:**

The wet mass of the tricipital muscles in the NMT-B group was significantly greater than the NMT-A, NMT-C, and model groups. In addition, MuRf-1 expression was significantly reduced in the NMT-B group compared with the NMT-A, NMT-C, and model groups.

**Conclusions:**

Elongating the nerve by NMT of 9% in rabbits decreased MuRf-1 expression and decelerated muscle atrophy in the subjects with sciatic nerve injury.

## Introduction

Nerves are special organs that exist in almost all tissues of the human body [1]. Compared with central nerves, a majority of peripheral nerves are more superficial and lack protection by bony structures, while a few of peripheral nerves are protected by bony structures, such as median never in carpal tunnel, they are more easily compressed than central nerves. Therefore, peripheral nerve injuries are a common clinical problem and often lead to long-term functional deficits [2]. Additionally, after peripheral nerve injury, the muscles without innervations begin to undergo atrophy and even irreversible degeneration. Therefore, the control atrophy and degeneration of denervated muscle has reemerged as a focus of study. MuRf-1 may act by degrading components of the contractile apparatus, its overexpression results in disruption of the muscle [3]. Therefore, inhibition of MuRf-1 may protect against denervated muscle atrophy.

Numerous studies have demonstrated that damaged nerves produce various pathophysiological responses, such as nerve tissue ischemia [4], axonal transport inhibition [5], and intraneural edema [6,7]. The neurodynamic mobilization technique (NMT) is a manual therapy method used by physiotherapists to assess and treat neuromuscular disorders. It includes gliding techniques and tensile techniques. Gliding techniques, or ‘sliders’, attempt to produce a sliding movement between neural structures and adjacent nonneural tissues and is executed in a non-provocative fashion. As the name implies, the purpose of neurodynamic tensile techniques are more aggressive than neurodynamic ‘sliders’. NMT is used to increase axonal transport and improve nerve conduction [8,9], and reduce the pressure existing within the nerve, thereby resulting in improved blood flow to the nerve. This increased flow may promote the regeneration and healing of the injured nerve [10]. A study also suggested that axon stretching may speed the rate of slow axonal transport and neuron growth [11]. Nerves possess viscoelastic properties [12,13], the reduction in stress is most rapid early in elongation. As a peripheral nerve elongates, its blood flow changes correspondingly. Clark et al. reported that nerve blood flow decreased approximately 50% when the nerve was elongated approximately 8% with substantial recovery, whereas an approximate 80% reduction in blood flow was observed after 15% elongation with minimal recovery. In addition, mechanical failure of the restored nerve was noted after 16 to 17% elongation [14]. Therefore, defining the tensile parameters of NMT is a key point in this study.

The purpose of this study is to determine the relationship between the lower limb joining angle and sciatic nerve elongation to define the tensile parameters of NMT and to observe the effect of NMT on tricipital muscle wet weight and MuRf-1 expression in rabbits with sciatic nerve injury. In view of the effects of NMT, we hypotheses that NMT decelerate muscle atrophy by restraining MuRf-1 expression.

## Material and methods

All experiments in this study were performed in compliance with the guidelines of the Second Affiliated Hospital of Heilongjiang University of Chinese medicine Institutional Laboratory Animal Care and Use Committee (ABX20130601A).

### To determine the relationship between the lower limb joint angle and sciatic nerve elongation to define the tensile parameters of NMT

**Figure 1:**
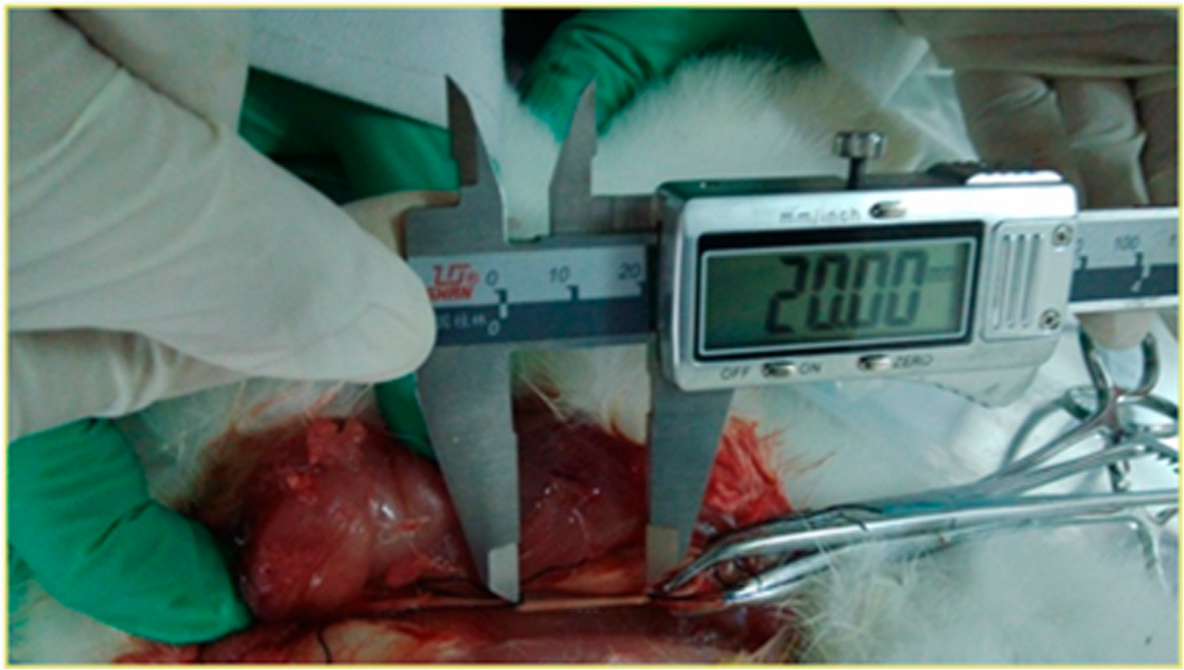
Original length of the sciatic nerve (mm).

**Figure 2:**
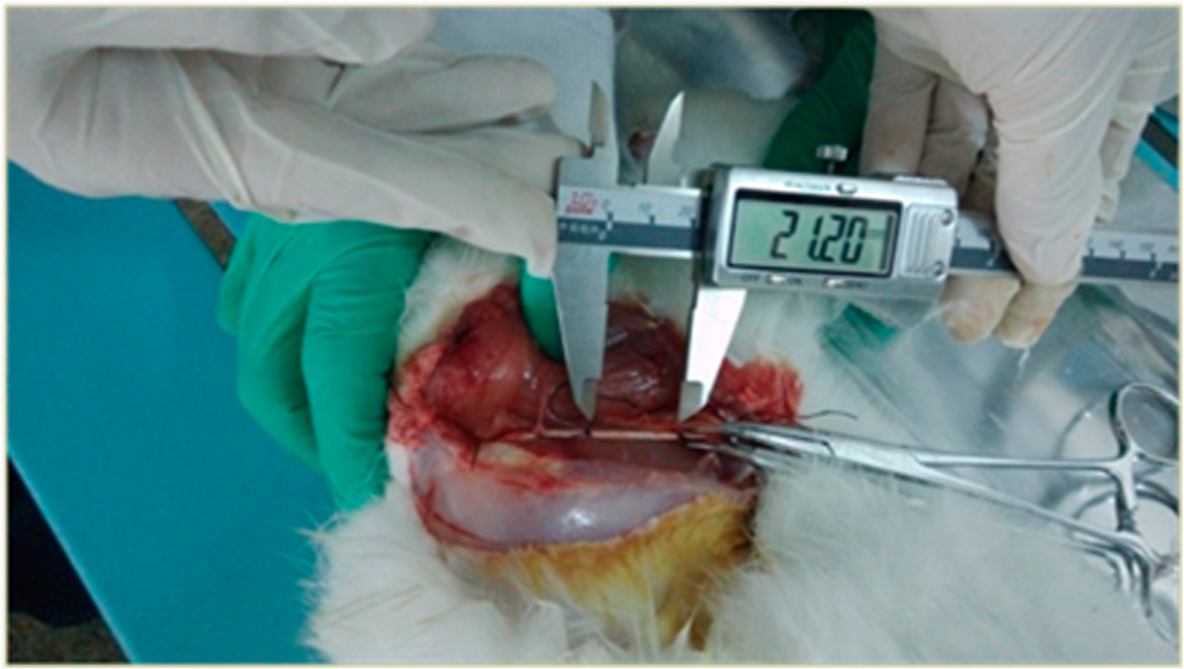
The sciatic nerve elongated 6% (mm).

**Figure 3:**
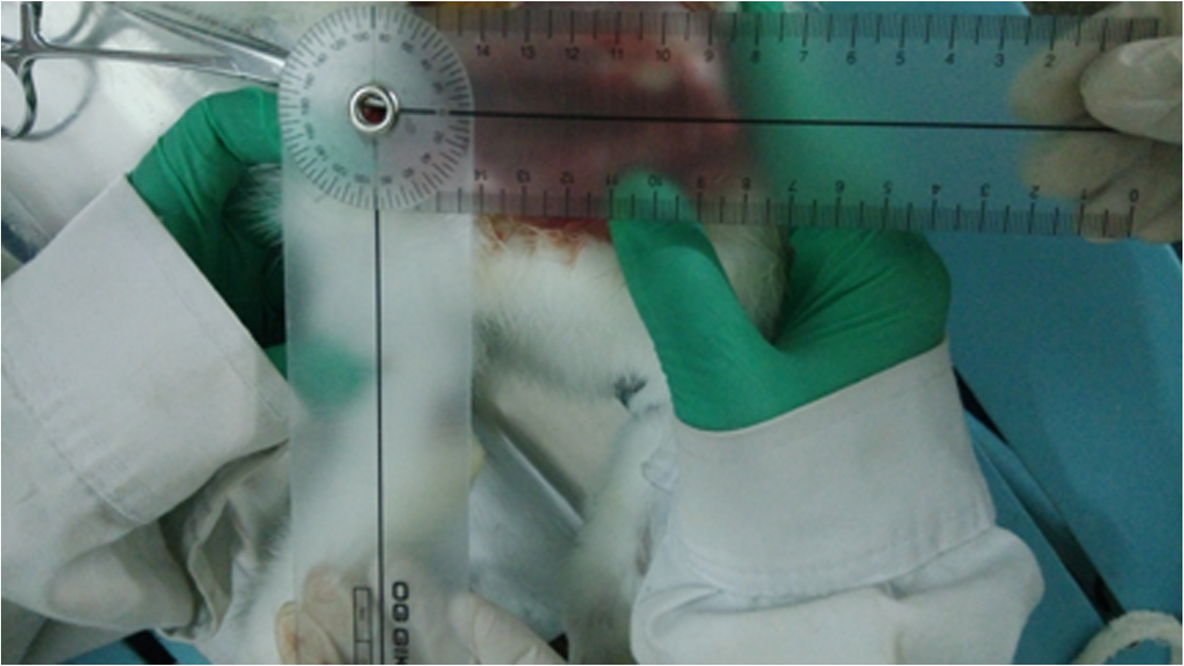
The knee joint angle of the sciatic nerve elongated by 6%.

Six 4 ~ 5-months old male adult Japanese white rabbits with a mean weight of 1.79 kg were supplied by Harbin Pacific Biological Pharmaceutical Co., LTD. [license SCXK (Hei), 2011–009]. After randomly numbering the rabbits from 1 to 6, they were anaesthetized with 1–2 ml/kg of 10% chloral hydrate via the ear marginal vein. A lateral approach was created, the attachment point of rectus femoris tendon on knee joint and the rectus femoris between up one third and middle were cut, and the sciatic nerve was exposed fully. Next, two surgical sutures, one was on 1 cm above lateral femoral epicondyle, another was on going up 20 mm apart, were fastened to the nerve trunk as markers. The rabbits were placed on the experiment table with their hip and knee joints at 45° and 30° angles, respectively. The knee joints of the rabbits were passively extended along the sciatic nerve. Meanwhile, Digital Vernier Calipers were placed on the nerve trunk with a precision of 0.01 mm. When the distance between the two markers in the nerve trunk was elongated to the appropriate length (nerve elongation 6%, 9% and 12% is 21.2 mm, 21.8 mm and 22.4 mm, respectively), the angle of the knee joint was measured. The mean of the knee joint angles of the six rabbits was used as the standard of NMT for different nerve elongations (Figures 1, 2, and 3). The relationship between the lower limb joint angle and sciatic nerve elongation is described as Table 1.

**Table 1:** **Relationship between the knee angle and sciatic nerve elongation**

**Nerve elongation**	**No 1**	**No 2**	**No 3**	**No 4**	**No 5**	**No 6**	**Mean**
6%	92°	90°	89°	90°	90°	90°	90.1°
9%	128°	135°	130°	133°	126°	128°	130°
12%	168°	174°	170°	169°	170°	168°	169.8°

### The effect of NMT on muscle atrophy and MuRf-1 expression

Thirty 4 ~ 5-months old male adult Japanese white rabbits with a mean weight of 1.81 kg were supplied by the same company. Animals were randomly assigned to the sham group (n = 6), model group (n = 6), NMT-A group (nerve elongation of 6%, n = 6), NMT-B group (nerve elongation of 9%, n = 6), and NMT-C group (nerve elongation of 12%, n = 6). The rabbits were anaesthetized, and the sciatic nerves were exposed as described above. The sham group was subjected to the same surgical procedure with sciatic nerve exposure but without crush injury. The hemostatic forceps was covered by an infusion set and clamped to the nerve trunk (1.5 cm above the lateral femoral epicondyle) with 3 buttons for 5 minutes; this caused approximately 2 mm of damage to the nerve.

The intervention methods for all groups as follows: sham and model groups: no intervention; NMT groups: based on the above experimental methods, intervention by NMT was conducted on the rabbits in the 3 groups 3 days after the operation. Before treatment, each rabbit was placed on the experiment table and with the lesioned limb hanging off the side of the table. Then the rabbit’s hip joint was flexed to 45°and the knee joint flexed to 30°. The knee joint was extended from 30° to 90° (NMT-A),30° to 130°(NMT-B), or 30° to 170° (NMT-C). The position of the limb was marked for each condition, as line 1,2,3 respectively. For rabbits in the NMT-A treatment group, a wooden blade was put on line 1, the researcher quickly extended the knee joint from start angle to line 1 and, then back to the start angle while an assistant stabilized the rabbit on the table. Rabbits in the NMT-B and NMT-C treatment groups received the same treatment as the NMT-A group, except a wooden blade was placed on line2 or 3 respectively. This NMT methodology produce ‘sliders’ which is non-provocative, because the damaged nerves are more mechanosensitive than healthy nerves. Each operation time was 1 s, and each relaxation time was 5 s; both were performed 10 times/group, 1group/day, and 6 days/week for a total treatment cycle duration of 4 weeks. At 4 weeks post-treatment, the bilateral tricipital muscles of the rabbits were excised. First, the tendons of gastrocnemius attached at medial and lateral condyles of femur were severed. Then the Achilles tendon was also cut. Next, the entire tricipital muscle was excised from the soft tissues connected with tibia and fibula. Finally, of the remaining soft tissue on the tricipital muscle was removed. The muscle wet mass was weighed using an electronic analytical balance with a precision of 0.1 g. Then, MuRf-1 expression in the triceps was measured by Western blot. Samples (200 μg) of skeletal muscle were ground and dissolved in the cold cracking protein liquid and centrifuged (12000 r/min, 10 min). A sample of the liquid mixed with protein (40 μg) was boiled (5 min). After electrophoresis, proteins were transferred to a nitrocellulose membrane, blocked with 1% nonfat milk, and incubated overnight at 4°C. The membranes were then washed with large volumes of TBST (3 times, for 10 min each), and exposed to a primary antibody overnight at 4°C. The membranes were then washed again (3 times, for 10 min each), then incubated with second antibody for 2 h at 37°C. Membranes were covered with ECL™ detection reagents and imaged (autoradiography film). The resulting bands on the films were analyzed to determine protein levels.

Statistical calculations were performed using SPSS 19.0 for Windows. The data of wet mass of muscles and MuRf-1 protein expression in this article were presented as means ± SD. All variables were tested for normal distribution using the Kolmogorov-Smirnov test (p > 0.05). One-way analysis of variance (ANOVA) followed by the LSD post-hoc test was used for the statistical analysis. A significant difference was defined as p ≤ 0.05.

## Results

### Wet mass of the tricipital muscles

**Figure 4:**
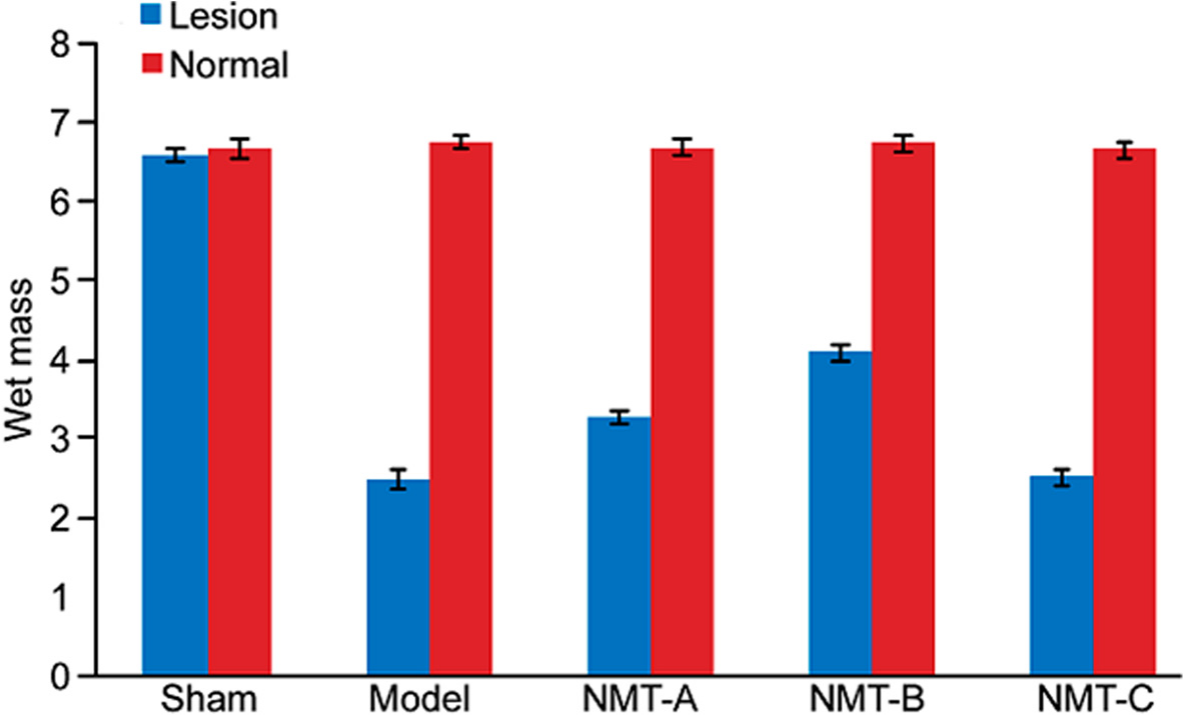
Wet mass of bilateral tricipital muscles. A significant reduction in the tricipital muscle wet mass was noted in the lesioned lateral muscle compared with the normal lateral muscle in all groups (p < 0.05) except the sham group (p > 0.05).In the lesioned lateral, the tricipital muscle wet mass in the NMT-B group was significantly increased compared with the NMT-A, NMT-C and model groups (p < 0.05).

The wet mass of the tricipital muscle was significantly less in the lesioned lateral muscle compared with the normal lateral muscle in all groups (p < 0.05) except the sham group (p = 0.289). Further, the tricipital muscle wet mass in the NMT-A and NMT-B groups was significantly increased compared to the model group (p < 0.05), whereas no significant difference was noted between the model and NMT-C groups (p > 0.05). The tricipital muscle wet mass in the NMT-B group was significantly higher than the NMT-A and NMT-C groups (Table 2 and Figure 4).

**Table 2:** **Comparisons of the wet mass of bilateral tricipital muscles(g,—X ± s)**

**Group**	**Lesion lateral**	**Normal lateral**
Sham group(n = 6)	6.57 ± 0.08	6.65 ± 0.14
Model group(n = 6)	2.48 ± 0.13*	6.73 ± 0.08
NMT-A group(n = 6)	3.27 ± 0.10#	6.67 ± 0.10
NMT-B group(n = 6)	4.07 ± 0.12▲	6.72 ± 0.10
NMT-C group(n = 6)	2.52 ± 0.10☆	6.65 ± 0.10

Lesioned lateral compared with normal lateral muscle, *#▲☆p < 0.05 in the model, NMT-B, NMT-A, and NMT-C group, respectively.

In the lesioned lateral, the wet mass of muscle in the model, NMT-B, NMT-A, and NMT-C group compared with in the sham group, *#▲☆p < 0.05; wet mass of muscle in the NMT-B and NMT-A compared with in the model group, #▲p < 0.05; the wet mass of muscle in the NMT-B compared with in the NMT-A and NMT-C group, # ☆p < 0.05.

### MuRf-1 protein analyses

**Figure 5:**
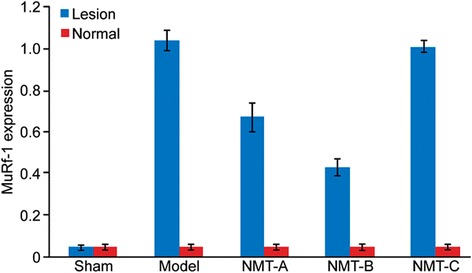
MuRf-1 expression in bilateral muscles. A significant increase in MuRf-1 protein expression was noted in the lesioned lateral muscle compared with the normal lateral muscle in all groups (p < 0.05) except the sham group (p > 0.05). In the lesioned lateral muscle, significantly reduced MuRf-1 protein expression was noted in the NMT-B group compared with the NMT-A, NMT-C and model groups.

**Figure 6:**
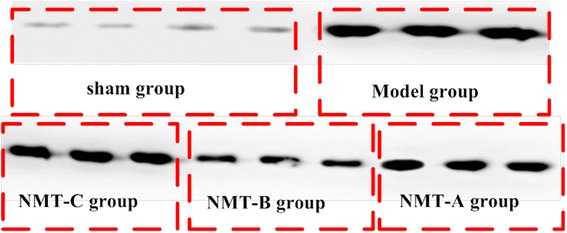
MuRf-1 expression in the triceps in lesion muscles was measured by Western blot. Levels of MuRf-1 in the NMT-B group were reduced compared with all groups except the sham group.

MuRf-1 protein expression increased in the lesioned lateral muscle compared with the normal lateral muscle in all groups (p < 0.05) except in the sham group (p = 0.197). In the lesioned lateral muscle, MuRf-1 protein expression was lower in the NMT-A and NMT-B groups compared to the model group (p < 0.05); whereas no significant difference was observed between the model and NMT-C groups (p > 0.05). MuRf-1 protein expression was reduced in the NMT-B group compared to the NMT-A and NMT-C groups (p < 0.05) (Table 3 and Figures 5 and 6).

**Table 3:** **Comparison of MuRf-1 protein expression in bilateral muscles(—X ± s)**

**Group**	**Lesion lateral**	**Normal lateral**
Sham group(n = 6)	0.05 ± 0.00	0.050 ± 0.000
Model group(n = 6)	1.04 ± 0.05*	0.052 ± 0.000
NMT-A group(n = 6)	0.67 ± 0.07#	0.051 ± 0.000
NMT-B group(n = 6)	0.43 ± 0.04▲	0.051 ± 0.000
NMT-C group(n = 6)	1.01 ± 0.03☆	0.050 ± 0.000

Lesioned lateral compared with normal lateral muscle, *#▲☆p < 0.05 in the model, NMT-B, NMT-A, and NMT-C group, respectively.

In the lesioned lateral, the MuRf-1 protein expression in the model, NMT-B, NMT-A, and NMT-C group compared with in the sham group, *#▲☆p < 0.05; the MuRf-1 protein expression in the NMT-B and NMT-A compared with in the model group, #▲p < 0.05; the MuRf-1 protein expression in the NMT-B compared with in the NMT-A and NMT-C group, # ☆p < 0.05.

These results indicate that NMT performed within the scope of safety may effectively reduce MuRf-1 protein expression and reduce muscle atrophy after nerve injury. Western blotting results indicate that the levels of MuRf-1 in the NMT-B group were reduced compared with other groups, except the sham group.

## Discussion

Previous studies of NMT have focused on neuropathic pain both in clinical trials and animal experiments [14-17]. Although these studies provide valuable information regarding peripheral nerve movement and strain, they provide no insight into the mechanisms responsible for the benefits of using NMT on denervated muscle. Moreover, the tensile parameters of NMT were not regulated in animal experiments. Driscoll et al. reported that nerve blood flow is reduced by approximately 70% and 78% after 8.8% and 16.1% strain, respectively; however, upon release of traction, blood flow exceeded baseline recordings, increasing to a mean of 151% of baseline blood flow after 8.8% strain and 82% of baseline after16.1% strain [12]. Others studies have also concluded that peripheral nerve elongation of less than 10% is safe and feasible [18]. Therefore, we chose a median elongation of 9%, and fluctuated down and up 3% to an elongation of 6% and 12%, respectively, to observe the influence of NMT on atrophy of denervated muscle. In this study, we report the relationship between the lower limb joint angle and sciatic nerve elongation. We also found different effects of NMT on muscle wet weight and MuRf-1 expression in rabbits with sciatic nerve injury.

Mild peripheral nerve injury may result in epineurial edema; with compression that is prolonged or of significant magnitude, the resulting endoneurial edema subsequently leads to fibrosis and adhesions [19]. Even worse, denervated muscle gradually atrophies and loses function. The pathogenesis of atrophy of denervated muscle is unclear. Nagata et al. suggested that a prolonged re-innervation process leads to skeletal muscle atrophy from disuse over time [20]. Fortunately, unlike the central nervous system (CNS), the peripheral nervous system (PNS) has an innate capacity to regenerate itself by innervating the muscle again [21]. Additionally, oxygen and nutrients inside the peripheral nerves are associated with nerve regeneration. However, vessels of peripheral nerve distribute particularly. Large blood vessels are primarily observed running longitudinally in the epineurial tissue, and branches of wide capillaries are present within the endoneurial compartment. NMT in the nervous system affects the availability of oxygen and nutrients as well as the ability to dispose of waste products, both of which are critical to nerve regeneration. Brown et al. found that the ‘pumping’ action that occurs with NMT may disperse the intraneural fluid and result in an altered intraneural pressure [22]. The above studies all demonstrated that NMT may improve nerve regeneration. Santiago et al. observed some reduction in muscle atrophy in rats that received an autograft and guide with adipose precursor cells compared with rats with untreated defects [23]. This finding suggested that nerve regeneration reduces muscle atrophy. Our results also indicate that in lateral lesions, the wet mass of the tricipital muscles in NMT-B group (9%) increased compared with the other groups. Whereas, the wet mass of the tricipital muscles in NMT-C (12%) decreased. These results are similar to those in the study by Driscoll et al. that indicated that nerve blood flow already exceeded baseline recordings after 8.8% strain and reduced after 16.1% strain when traction was released [12]. Therefore, we believe that NMT of nerves elongated by approximately 9% potentially aids in nerve regeneration and reduces muscle atrophy, whereas elongation greater than 12% is potentially harmful to the nerve. *In vivo* studies in a MuRf-1deficient mouse model demonstrated that limb muscle degeneration after a sciatic nerve lesion was decelerated by 36% [24]. MuRf-1 expression is negatively correlated to the degree of muscle atrophy. The Western blot results in our study indicated that MuRf-1 expression in the NMT-B group (9%) was lower than in all other groups except the sham group. Gumucio et al. reported the modulation of its expression via physical activity has the potential to prevent or reverse muscle atrophy [25]. Similarly, this study demonstrated that NMT resulting in nerve elongation of approximately 9% is an effective intervention method to decreased MuRf-1 expression, thereby decelerating muscle atrophy.

## Conclusions

Taken all together, our study for the first time defined the tensile parameters of NMT and demonstrated that elongating the nerve by NMT of approximately 9% in rabbits decreases MuRf-1 expression and decelerates muscle atrophy in the subjects with sciatic nerve injury. However, the current results are limited by the absence of direct evidence regarding the path by which NMT reduces MuRf-1 expression and decelerates muscle atrophy. Further studies will be required to identify the mechanism by which NMT reverses denervation muscle atrophy via signaling pathways.
